# Illusion for Airborne Sound Source by a Closed Layer with Subwavelength Thickness

**DOI:** 10.1038/s41598-018-38424-3

**Published:** 2019-02-11

**Authors:** Xu-Dong Fan, Bin Liang, Jing Yang, Jian-Chun Cheng

**Affiliations:** 1 Collaborative Innovation Center of Advanced Microstructures and Key Laboratory of Modern Acoustics, MOE, Institute of Acoustics, Department of Physics, Nanjing University, Nanjing, 210093 P. R. China; 2 National Center for Physical Acoustics and Department of Physics and Astronomy, University of Mississippi, University, Mississippi, 38677 USA

## Abstract

The past decade witnesses considerable efforts to design acoustic illusion cloak that produces the desired scattered field for a specific object illuminated by an external field. Yet the possibility of generating acoustic illusion directly for a sound source still remains unexplored despite the great fundamental and practical significance, and previous transformation acoustics-based designs need to have bulky sizes in terms of working wavelength. Here we propose to produce arbitrary illusion for an airborne sound source with no need to resort to coordinate transformation method. Based on an inherently different mechanism that uses acoustic metasurface to provide azimuthally-dependent local phase delay to the radiated wavefront, we shrink the thickness of the single layer enclosing the source to subwavelength scale without modulating the shape of layer. The performance of our scheme is demonstrated via distinct phenomena of virtually shifting the source location and introducing angular momentum. Numerical results verify our theoretical predictions, showing the extraordinary capability of the presented device to freely manipulate the radiation pattern of a simplest point source, making it acoustically appearing like another arbitrarily complicated source. Our findings open new avenues to the design and application of acoustic illusion devices and may have deep implications in many diverse fields such as architectural acoustics and biomedical engineering.

## Introduction

The interest in acoustically concealing and even camouflaging objects, which also represents one of the fundamental demands of various fields ranging from underwater acoustics to ultrasound biomedical imaging to architectural design, has grown into a field of intense research^1–31^. During the past few years, great efforts have been dedicated to the investigation on mechanisms for manipulating the scattered acoustic waves generated by an object interacting with incident waves, giving rise to conceptual devices of acoustic invisibility cloak for cancelling scattering effect followed by illusion cloaks that further enable freely changing the scattered field^1–19^. Unlike its electromagnetic counterpart composed of a pair of complimentary media with double negative effective parameters and working for an object in free space, the first acoustic illusion cloak is made of homogeneous positive-indexed media and is able to change the acoustical appearance of any object near an arbitrary curved boundary, making the experimental realization and characterization possible^20^. Such a two dimensional structure is later extended, both theoretically and experimentally, to a three dimensional version with the ability to work omnidirectionally regardless of the incident angle of the impinging acoustic wave^21^. Special types of acoustic illusions have also been generated by using metamaterials to virtually rotate the wavefront by a predesigned angle for sources inside or outside of the rotator^22,23^. However, the existing illusion cloaks need to rely on transformation acoustics techniques and therefore have to be implemented with bulky structures much thicker than the working wavelength, which would substantially hinder their real-world applications that usually demand for miniaturization and integration of devices. More importantly, the previous acoustic illusions are generated for reflected acoustic waves on an object illuminated by an external acoustic field. Yet the mechanism for producing acoustic illusion directly for an airborne sound source itself still remains elusive despite its significance in physics and application potentials for a great variety of scenarios.

In this article, we make an attempt to address these issues and realize illusion phenomenon for a simple point source by using a thin-layered enclosure that offers free manipulation of spatial field radiated by the emitting source itself. This extraordinary effect is enabled by a distinct mechanism that uses a circularly closed metasurface providing judiciously-engineered angular distribution of local discontinuity of propagating phase, instead of relying on transformation acoustics technique as in earlier illusion cloak designs, to straightforwardly modulate the wavefront of the radiated wave into the desired shape. We also present a practical implementation of our design by designing a specific kind of metamaterials comprising an open channel and a series connection of resonators, which allows control of the propagation phase within a full 2*π* range while keeping near unity transmission efficiency. Thanks to the proposed mechanism for production of acoustic illusion of sound source, the resulting device has compact structure with subwavelength thickness, regular geometry independent of the illusion type, high transparency to the radiation energy, flexibility of generating arbitrary illusion effects by adjusting the structural parameters distribution along the azimuthal direction. We use numerical simulations to verify the effectiveness of our scheme and choose two particular cases of source shifter and helical wave generator to demonstrate the functionality and versatility of the designed device.

## Results

### The mechanism of sound source illusion

**Figure 1:**
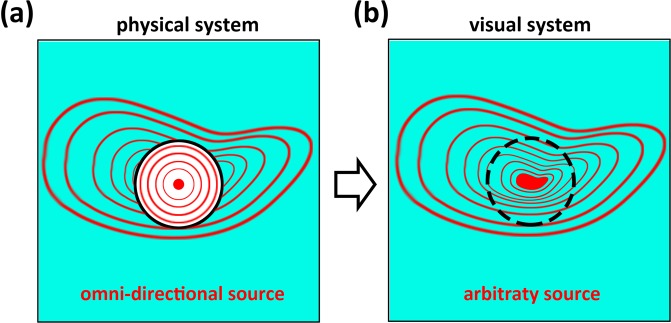
Schematic of the design for producing an illusion for airborne sound source. (**a**) Sound field outside the closed subwavelength layer (the black circle) surrounding a simple point source is identical to (**b**) the one generated by an arbitrary complicated source.

Figure 1 schematically shows our proposed mechanism for producing illusion effect for an airborne sound source. With no loss of generality, in this study we consider a two dimensional system and a simplest possible point source emitting acoustic energy equally in all directions. Unlike the conventional acoustic illusion phenomena characterized by the modulation of spatial distribution of acoustic energy scattered by another object as illuminated by an external source, the concept of source illusion we attempt to realize here refers to an all-angle modulation of the wave field from a source^13^. Our mechanism is that, with no need to depend on coordinate transformation-based schemes that mathematically design special parameter distribution and hence must have a bulky structure for controlling the way how the propagation trajectory is bent, we use a closed layer with subwavelength thickness to provide an azimuthally-dependent phase delay without attenuating the amplitude appreciably as the emitted wave traverses it, as depicted in Fig. 1. This mechanism also exempts from modification of the outer shape of the designed device, which would be necessary for the transformation acoustics-based schemes for avoiding extreme and inhomogeneous acoustical parameters and may result in special cloak shapes with uneven thickness such as the bowl-like structure in the previous works^20,21^. We thereby break through the limits in dimension and shape of device and enable the production of a different type of illusion effect that directly changes the acoustical appearance of the source. In other words, our proposed mechanism is capable of making a simple point source “becomes” another arbitrarily complicated source when detected by an observer at far-field region.

In our design, different types of acoustic source illusions are realized by simply engineering the phase profile on the surface of the designed device chosen to have an axisymmetric circular shape for simplicity as depicted in Fig. 1. In the current study, we choose two representative examples to demonstrate the performance of our design via production of source illusions including virtual shift of source location and generation of helical wave.

### Virtual shift of source location

**Figure 2:**
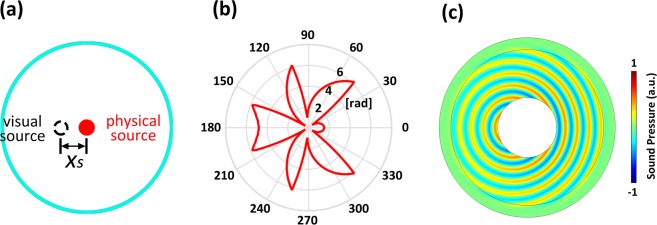
(**a**) Schematic of the source shifter. (**b**) Desired phase profile versus azimuthal angles. (**c**) Simulated result with theoretical parameters.

In order to design a source shifter capable of virtually moving the location of source by a certain distance, *i.e*., from the origin (0, 0) to (*x*_*s*_, *y*_*s*_), the azimuthal distribution of the phase shift should satisfy

1 $$\phi (\theta )=k(\sqrt{{(r\cos \theta -{x}_{s})}^{2}+{(r\sin \theta -{y}_{s})}^{2}}-r)$$

which comes from the relative geometry relation between the locations of meta-structure and the virtual source, where $k=2\pi /\lambda$ refers to the wave number, *λ* is the wavelength, *r* is the radius of the closed layer and *θ* is the azimuthal angle. The effectiveness of the designed source shifter is verified numerically and the typical results are shown in Fig. 2. In Fig. 2, the parameters are chosen as *λ* = 0.02 m, *r* = 2*λ*, *x*_*s*_ = −*λ* and *y*_*s*_ = 0. As demonstrated by the numerical results, the acoustic wave field outside the designed device remains the cylindrical form for which the wavefront is still axisymmetric but the symmetry center is no longer located at the origin (0, 0). For an observer outside, therefore, it seems that the acoustic energy is radiated omnidirectionally into all directions from a point source situated at the predesigned location of (*x*_*s*_, *y*_*s*_), giving rise to the desired illusion effect of source shift that virtually shifts the source position by a distance that can be freely adjusted by tuning the phase profile on the surface of device.

### Generation of radiation pattern of helical wave

In what follows we will further demonstrate the capability of our proposed design to transform the pattern of radiation field of a point source, for producing the illusion effect that makes it acoustically “become” a more complicated one. Here we choose to realize a helical wave generator that is able to introduce orbital angular momentum into an acoustic system with perfect axisymmetric geometry. To this end, the desired phase shift provided by the designed device needs to follow a particular angular dependence as given below:

2 $$\phi (\theta )+N\theta +{\phi }_{0}+k(\sqrt{{x}^{2}+{y}^{2}}-r+\sqrt{{(r\cos \theta -{x}_{0})}^{2}+{(r\sin \theta -{y}_{0})}^{2}})$$

where *N* refers to the additional angular momentum, ${\phi }_{0}$ is the initial phase and the third term comes from the relative geometry relation between the locations of meta-structure and the source with (*x*_0_, *y*_0_) being the position of physical source.

**Figure 3:**
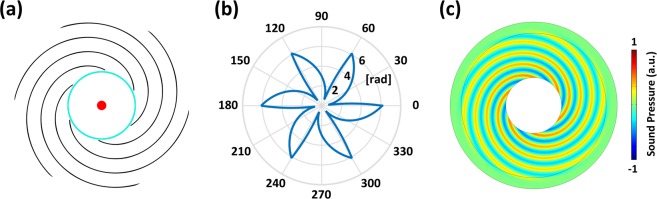
(**a**) Schematic of the source converter. (**b**) Desired phase profile versus azimuthal angles. (**c**) Simulated result with theoretical parameters.

The corresponding numerical results are plotted in Fig. 3, in which the parameters are chosen as *N* = 6, ${\phi }_{0}=0$ and $({x}_{0},\,{y}_{0})=(0,\,0)$. As shown by the results, the acoustic wave radiated by the simple point source, which will be cylindrical waves in the absence of the proposed device, now has a helical-like wavefront after transmitting through the device with subwavelength thickness, during which the propagation phases at different azimuthal angles are modulated differently according to Eq. (2). As a consequence, the source perceived by the observer outside will be a point source with spiral wavefront, with the numbers of branches equal to the predesigned value of *N* = 6, which indicates that the propagation phase undergoes 2*πN* change in an annular loop and proves that the angular momentum imparted to the original point source is exactly *N* as expected.

**Figure 4:**
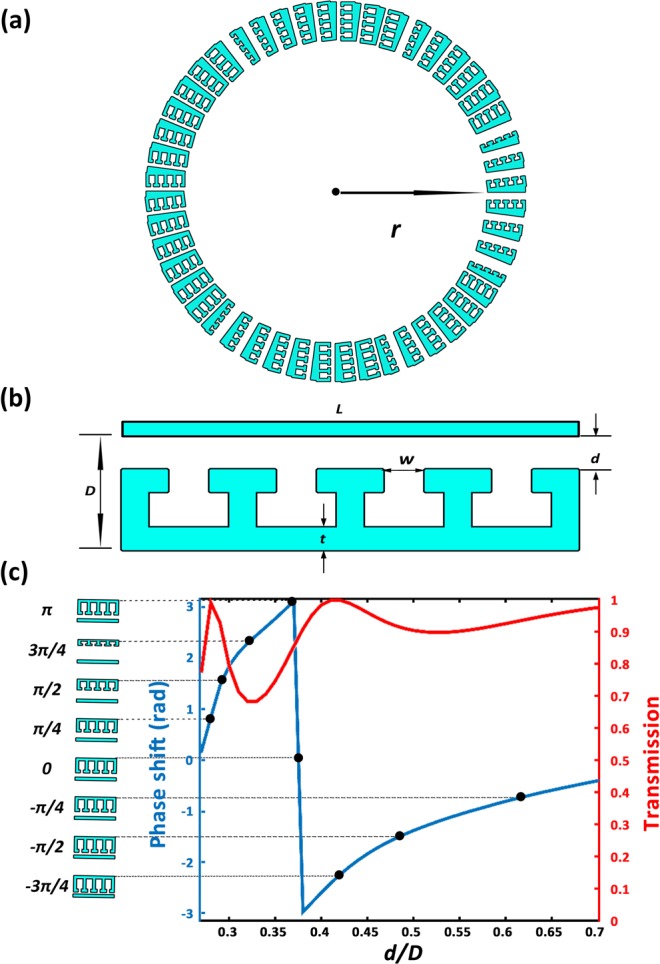
(**a,b**) Schematics of (**a**) the structure for source shifter and (**b**) of an individual unit cell. (**c**) Simulated transmission coefficient and phase shift of transmitted wave through individual units with different values of d/D. The eight dots mark the discrete phases provided by the eight particular unit cells to be used in the numerical simulations.

Our proposed scheme can be implemented by acoustic metasurfaces offering great flexibility of modulating propagating phase with vanishing thickness and planar geometry^24,32,33^. The configuration of the metasurface used in the current study is schematically illustrated in Fig. 4a, which is composed of 48 fan-like unit cells in Fig. 4b with each having a hybrid structure formed by coupling a straight tube with a series connection of four cavities acting as acoustic resonators. Parameters are chosen as *L* = *λ*/2, *D* = *λ*/4, *t* = *λ*/40, and *w* = 2*t*. The effective acoustical parameters are retrieved via numerical simulations and we plot the typical results of transmission coefficient and phase shift for an individual unit cell in Fig. 4c. The numerical results show that such type of hybrid metastructure can freely tune the propagation phase within the full 2*π* range yet keep a near unity transmission despite subwavelength dimensions both in the radial and azimuthal directions, which makes it a good candidate for composing acoustic metasurface with high efficiency and fine phase resolution as a practical implementation of our designed source illusion device.

**Figure 5:**
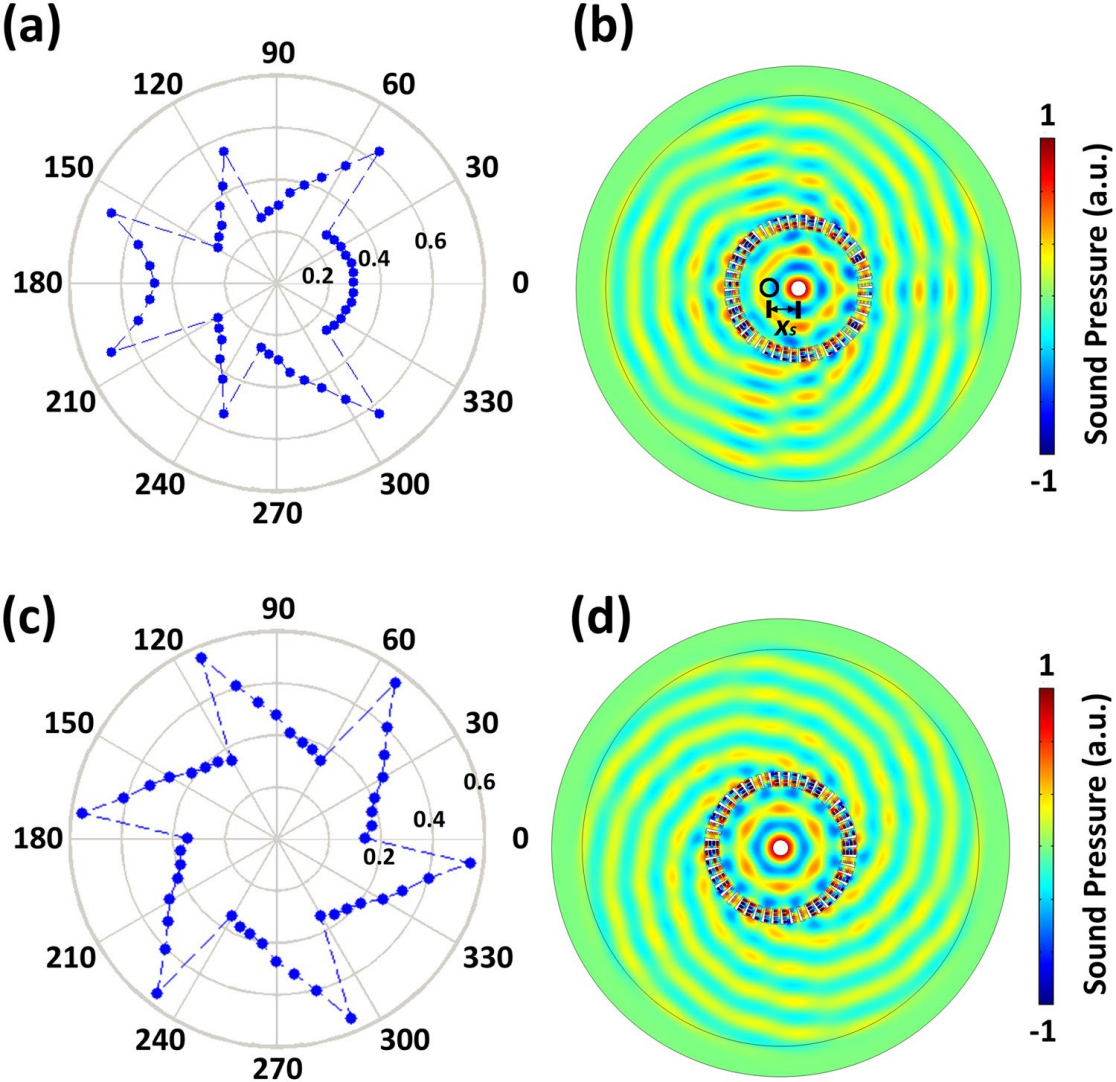
(**a,c**) Discrete parameter profile of *d*/*D* provided by the practical devices for (**a**) a source shifter and (**c**) a source converter versus azimuthal angles. (**b,d**) Simulated pressure field for (**b**) source shifter and (**d**) source converter implemented by practical meta-structure.

Next we use numerical simulations to demonstrate the performance of the metasurfaces-based implementation of our designed device and depict the typical results in Fig. 5. Figure 5a shows the value of *d*/*D* as a function of azimuthal angle which is predicted by Eq. (1) and Fig. 4c. The generated acoustic field by a point source closed by such a source shifter is plotted in Fig. 5b. From the simulated spatial distribution of acoustic pressure it is apparent that the acoustic field produced by the practical device implemented by properly designed metasurfaces is nearly identical with the results obtained for the idealized design (Fig. 2), which is guaranteed by the high transmission efficiency and fine phase-profile resolution offered by our proposed hybrid metastructure. It is therefore reasonable to identify such device implemented based on metasurfaces as an effective source shifter for airborne sound. Similarly, we simulate the radiated field of a point source located at the origin in the presence of a practical implementation of a source converter and plot in Fig. 4c,d the numerical results, together with the parameter distribution. The results also agree quite well with the fore-mentioned theoretical predictions, with both proving that such metasurface-based device can satisfyingly mimic a source converter capable of imparting a simple point source with the desired angular momentum precisely, except for slight fluctuations in the pressure amplitude due to the fact that the transmission in practical metastructure cannot reach 1 exactly.

## Discussion

In summary, we propose the concept of source illusion for airborne sound and use modulated angular distribution of phase discontinuity to directly engineer the wavefront transmitted from inside to outside, downscaling the layer thickness of device to subwavelength regime. We have also demonstrated a particular implementation by using acoustic metasurface composing of an array of hybrid metastructure proven to have high transmission and full phase control simultaneously. In comparison with the previous transformation acoustics-based designs with bulky structures for producing illusion by changing the scattered field on an object illuminated by external wave, our presented mechanism for the first time enables the production of source illusion effect that directly changes the radiation field pattern from a simple source arbitrarily, and also bears the advantages of compact size, high efficiency, and large flexibility. The realization of source illusion for airborne sound opens up new possibilities for the design and application of acoustic illusion devices, and may find important uses in a great variety of scenarios that demand for special manipulations of radiation field such as in audible sound control, biomedical application, underwater camouflage and so on.

## Methods

Throughout the paper, the numerical simulations are performed by the finite element method based on commercial software COMSOL Multiphysics. The solid materials applied in the simulations is chosen as Acrylonitrile-Butadiene-Styrene (ABS) plastic for which the sound speed and mass density are $c=2700\,{\rm{m}}/{\rm{s}}$ and $\rho =1180\,{\rm{kg}}/{{\rm{m}}}^{3}$ respectively. The standard parameters used for air under an ambient pressure of 1 atm at 20 °C are mass density ${\rho }_{0}=1.21\,kg\cdot {m}^{-3}$ and sound speed ${c}_{0}=343\,m\cdot {s}^{-1}$.
